# Anesthetic Management in a Child With Citrullinemia: A Case Report

**DOI:** 10.5812/aapm.21791

**Published:** 2014-08-13

**Authors:** Mohammad Gharavifard, Alireza Sabzevari, Reza Eslami

**Affiliations:** 1 Department of Anesthesiology, Faculty of Medicine, Mashhad University of Medical Sciences, Mashhad, Iran; 2 Surgical Oncology Research Center, Mashhad University of Medical Sciences, Mashhad, Iran

**Keywords:** Citrullinemia, Urea Cycle Disorders, Inborn, Hyperammonemia, Anesthesia

## Abstract

**Introduction:**

Citrullinemia is a defect in the urea cycle that causes ammonia to accumulate in the blood. We describe the anesthetic management of a patient with citrullinemia, who experienced an unexpected 10 day hospital admission.

**Case Presentation:**

We anesthetized a 3.5 year-old boy with citrullinemia who was scheduled for a dentistry procedure. Perioperative precautions included minimizing fasting period, hypothermia prevention, relieving anxiety and pain, perioperative infusion of D10W and benzoate sodium, as well as a pediatric endocrinology consultation. The operation lasted 4 hours and its course was uneventful. He had a delayed recovery from anesthesia and was discharged from hospital after 10 days.

**Conclusions:**

General anesthesia and surgery can be a risk factor for exacerbating the course of the disease in patients with citrullinemia. It appears that administering short acting sedatives and analgesics in these patients would be of more benefit. Further studies are required to identify a safe method for anesthesia in citrullinemia.

## 1. Introduction

Citrullinemia is one of the urea cycle disorders that lead to the accumulation of nitrogen as ammonia, alanine, glutamate, and other intermediate metabolites, as well as arginine synthesis disorder (1, 2). This autosomal recessive disorder includes an acute neonatal type I (the “classic” form), and adult onset type II citrullinemia hyperammonemia (1, 3, 4).

Citrin deficiency is one of the most common urea cycle disorders in the world, while the exact effect of the perioperative anesthetic regimen on postoperative metabolic changes, side effects, and length of hospital stay is not well understood. Most information is derived from individual case reports (1, 5). This means that early diagnosis and management of the side effects are two key factors for controlling postoperative conditions in these patients. The following report describes a 3.5 year-old child who showed a delayed recovery after general anesthesia for dentistry procedure.

## 2. Case Presentation

A 3.5 year-old boy, weighing 14 kg, who was a known case of citrullinemia, was referred for a dentistry procedure under general anesthesia. Familial history revealed death of a sibling at the second day of life and past drug history included: Sodium benzoate (2 g/q6h), L-arginine (250 mg/q12h), L-carnitine (500 mg/q8h), ranitidine (45 mg/q12h), syrup of calcium, multivitamins plus zinc, and metronidazole (10 d/q month). The laboratory findings are summarized in Table 1. Physical examination revealed no significant findings. The American Society of Anesthesiologists (ASA) physical classification of the patient was class 2. The patient received sodium benzoate (200 mg/kg) 3 hours before surgery, as recommended by the pediatric endocrinologist. The fasting time was minimal and the operation was planned for the early morning. To separate the child from the parents and to insert an intravenous catheter, we used sevoflorane (8%) and a mixture of N_2_O (60%) and O_2_ (40%) via face mask. The anesthesia was induced with propofol (2 mg/kg), fentanyl (3 µg/kg) and cisatracurium (0.1 mg/kg). Fentanyl and cisatracurium were repeated with 1/5 dose as needed. Normal saline plus 10% dextrose in water (D10W) solution was infused during the operation. The patient was monitored by pulse oximetry, capnography, a non-invasive blood pressure device, and nasopharynx thermometer. Blood glucose was tested every hour. To prevent postoperative pain, morphine (100 µg/kg) was administered one hour prior to the conclusion of surgery. The surgery and general anesthesia were ended uneventfully after four hours. Spontaneous respiration returned and complete reversal of neuromuscular block was achieved by administering neostigmine. However, regaining consciousness was delayed. Low dose naloxone was administered incrementally due to significant miosis. The patient still remained unconsciousness 2 hours after the operation. At this time, blood was sampled for determination of plasma concentrations of ammonia, glucose, urea, creatinine, Na, K, and Ca. The results are shown in Table 2.

Then, we began infusion of sodium benzoate (200 mg/kg). No obvious changes in consciousness of the child occurred. Eventually, the patient opened his eyes four hours after the end of surgery. However, the patient was unable to maintain his head posture and seemed ataxic. He was transferred to the pediatric intensive care unit (PICU) and was discharged 10 days later after gradually regaining his complete health.

**Table 1.** Preoperative Laboratory Test Results

Variable	Preoperative
**Ammonium, μmol/dL**	34
**WBC, 10** ^**3**^ **/uL**	7.2
**RBC, 10** ^**6**^ **/uL**	4.4
**Hb, g/dL**	11.2
**HCT, %**	32.5
**pH**	7.42
**Spo**_**2,**_ **%**	98
**PaCo**_**2,**_ ** mmHg**	29.8
**BE, mEq/L**	-5.2
**PaO**_**2,**_ ** mmHg**	310
**Peripheral blood smear**	Hypochromic and microcytic
**Glucose, mg/dL**	108
**Cr, g/dL**	0.7
**Ca, mg/dL**	8.9
**Na, mEq/L**	140
**K, mEq/L**	4.1
**BUN, mg/dL**	11

**Table 2.** Postoperative Laboratory Test Results

Variable	Postoperative
**Ammonium, μmol/dL**	52.3
**WBC, 10** ^**3**^ **/uL**	7.8
**RBC, 10** ^**6**^ **/uL**	4.52
**Hb, g/dL**	11
**HCT, %**	32
**pH**	7.36
**SpO**_**2,**_ ** %**	98
**PaCO**_**2,**_ ** mmHg**	38
**BE, mEq/L**	-4.5
**PaO**_**2,**_ ** mmHg**	273
**Peripheral blood smear**	Hypochromic and microcytic
**Glucose, mg/dL**	180
**Cr, g/dL**	0.7
**Ca,mg/dL**	8.5
**Na, mEq/L**	142
**K, mEq/L**	4.3
**BUN, mg/dL**	8

## 3. Discussion

There are many reasons for delayed emergence after general anesthesia, which include metabolic and non-metabolic causes. Nevertheless, general anesthesia by propofol and fentanyl, which we also used, is a good choice for painful procedures in children (6). The situation is further complicated in a patient with preexisting metabolic disorders such as a case of citrullinemia.

Although this patient was a known case, yet most neonatal citrullinemia patients are asymptomatic at birth. Therefore, family history may be helpful in diagnosing the cause of postoperative delayed emergence and impaired consciousness in any neonate undergoing surgery. In combination with positive laboratory results, a full medical history may indicate a probable citrullinemia disorder in an infant. A family history of abortion has been reported in other citrullinemia case reports (7).

Postoperative prolonged unconsciousness might be the result of the ammonia level, blood glucose or a side effect of the anesthetic agents administered. It should be noted that in times of stress such as surgery, sepsis, dehydration, anesthesia, fasting, high protein diet, and intravenous feeding, ammonia accumulation may occur due to protein degradation (8). Despite impaired consciousness has been reported with a higher elevation of serum ammonia, the blood ammonia level did not always correlate with the severity of disturbed consciousness (9). In the case presented here, although the preoperative ammonia level was normal, the post-operative level of ammonia was increased a little. So, it seems reasonable to measure the ammonia level before and after the operation.

Our treatment for severe miosis (by naloxone) did not improve the patient's consciousness. In this respect, the infusion of sodium benzoate followed by intralipid may lead to an improvement in the patient's reflexes and consciousness. However we cannot substantiate a causality relationship. In this case, the residual effect of morphine and other drugs administered may be responsible for delayed awakening, though there is no clear evidence for such an effect. According to pediatric neurologists’ consultations, patient’s metabolic disease was the cause of postoperative ataxia. So, there was no need to further attempts and just the patient was managed conservatively in the PICU.

The reports of classic citrullinemia, which are often asymptomatic, highlight the need for appropriate guidelines for postoperative care and an update to the current guidelines for anesthesia protocols in hyperammonemia patients. It appears reasonable not to operate on patients with the severe forms of citrullinemia unless the necessary conditions are met.

In conclusion, if general anesthesia is indicated for patients with a severe form of citrullinemia, drugs that are minimally metabolized, and those with a short acting effect should be cautiously administered. Intraoperative glucose and ammonia blood level measurements can be beneficial. Infusion of sodium benzoate during prolonged operations may be helpful in rapid emergence of the patient to prevent citrullinemia complications. Further studies are required to identify the best protocol for anesthesia in citrullinemia.
